# Osteoporotic vertebral fracture misdiagnosed as “normal postoperative phenomenon” in post decompression surgery: a case report

**DOI:** 10.1186/s12891-020-03904-z

**Published:** 2021-01-15

**Authors:** Li-sheng Hou, Dong Zhang, Feng Ge, Hai-feng Li, Tian-jun Gao

**Affiliations:** Department of Orthopedic Surgery, The Sixth Medical Center of PLA General Hospital, Fucheng Road, Haidian District, Beijing, 100048 China

**Keywords:** Percutaneous transforaminal endoscopic decompression, Normal postoperative phenomenon, Misdiagnosis, Osteoporotic vertebral fracture

## Abstract

**Background:**

Previous research and published literature indicate that some patients with spinal diseases who underwent percutaneous transforaminal endoscopic decompression (PTED) still suffer some discomfort in the early recovery stage in the form of pain, stiffness, and swelling. These are usually considered minor residual symptoms or normal postoperative phenomenon (NPF) in the clinic, occur frequently, and are acknowledged by surgeons worldwide.

To the best of our knowledge, we report the first case of a patient who had an osteoporotic vertebral fracture (OVF) misdiagnosed as NPF after she underwent PTED as a result of lumbar disc herniation (LDH).

**Case presentation:**

A 71-year-old female with Parkinson’s disease who presented with lower back pain radiating to the legs was diagnosed as LDH in L4–5, after which a PTED of L4–5 was performed, with temporary alleviation of symptoms. However, severe lower back pain recurred. Unfortunately, the recurred pain initially misdiagnosed as NPF, in fact, was finally confirmed to be OVF by CT-scan. OVF in the early stage of post-PTED seldom occurs and is rarely reported in the literature.

With a percutaneous vertebroplasty, the pain was significantly relieved, and she resumed walking. After 36-weeks of follow-up, the pain improved satisfactorily.

**Conclusion:**

Doctors should not immediately diagnose a relapse of back pain following PTED as NPF, and hands-on careful physical and imaging examinations are necessary to manage recurring pain rightly and timely.

## Background and introduction

Currently, percutaneous transforaminal endoscopic decompression (PTED) is being widely used to treat degenerative lumbar disorders due to less iatrogenic trauma and quick recovery [1, 2]. However, “quick recovery” does not immediately lead to the resolution of symptoms after PTED. On the basis of classic literature, experts’ opinions and surgeons’ experience, occasionally, in postoperative stage, some patients still endure discomfort, such as pain, stiffness, and motion limitation, usually recognized as residual symptoms or normal postoperative phenomena (NPF) [3, 4]. Generally, NPF occurs within 3 to 8 weeks postoperatively or later and is mostly treated with conservative treatment including non-steroidal anti-inflammatory drugs (NSAIDs) and physical factor treatments, such as in the forms of heat therapy and massage.

Parkinson’s disease (PD) is a neurodegenerative disorder that commonly occurs in the elderly, has a tendency to reduce bone mass, and is closely associated with osteoporotic vertebral fracture (OVF), which might be missed during clinical check-up or neglected as soft tissue impairment if in the absence of a clear trauma history [5]. This circumstance might be very common clinically but is frequently missed.

Here, we present a rare case of a patient with PD post-PTED for degenerative lumbar disc herniation (LDH) who developed OVF during the early recovery stages, which seldom occurs and is rarely reported in literature. Initially, the OVF was misdiagnosed as NPF following PTED. Once diagnosed correctly, a percutaneous vertebroplasty (PVP) alleviated the symptoms dramatically.

## Case presentation

### History and examinations

A 71-year-old female was admitted to our hospital with a complaint of worsening pain in waist, which radiated to her left leg with feeble strength for 2 months. The visual analogue scale (VAS) score for pain was 5 points for the waist and 8 for the left leg. She had orally taken Eperisone Hydrochloride tablets, Aescuven Forte, and Mecobalamin, however, with no relief.

She had PD for 11 years and maintained on Madopar, Adamantane and Sinemet, which was unluckily valid last year. Hence, 1 year ago, she underwent deep brain electrode implantation (DBEI) for PD. Five years ago, she underwent a PVP for compressive fracture of T11 after a fall. Since then, she has been taking calcium and vitamin D supplements, but not regularly.

Upon admission, a physical examination showed that she was unresponsive to questions, had intermittent tremors, truncal rigidity, and bradykinesia. The patient also had radiating pain and numbness on anterolateral and medial sides of the left leg and the lateral side of the left dorsal foot. She tested positive for the straight-leg-raising test (40 degrees) and negative for the femoral nerve stretch test. Her muscle strength of the left L5 myotome was slightly decreased (grade IV-). She scored a total of 224 points on the Short- Form-36-questionnaire (SF-36) and 10 points on the Japanese orthopedic association (JOA) score.

**Fig. 1:**
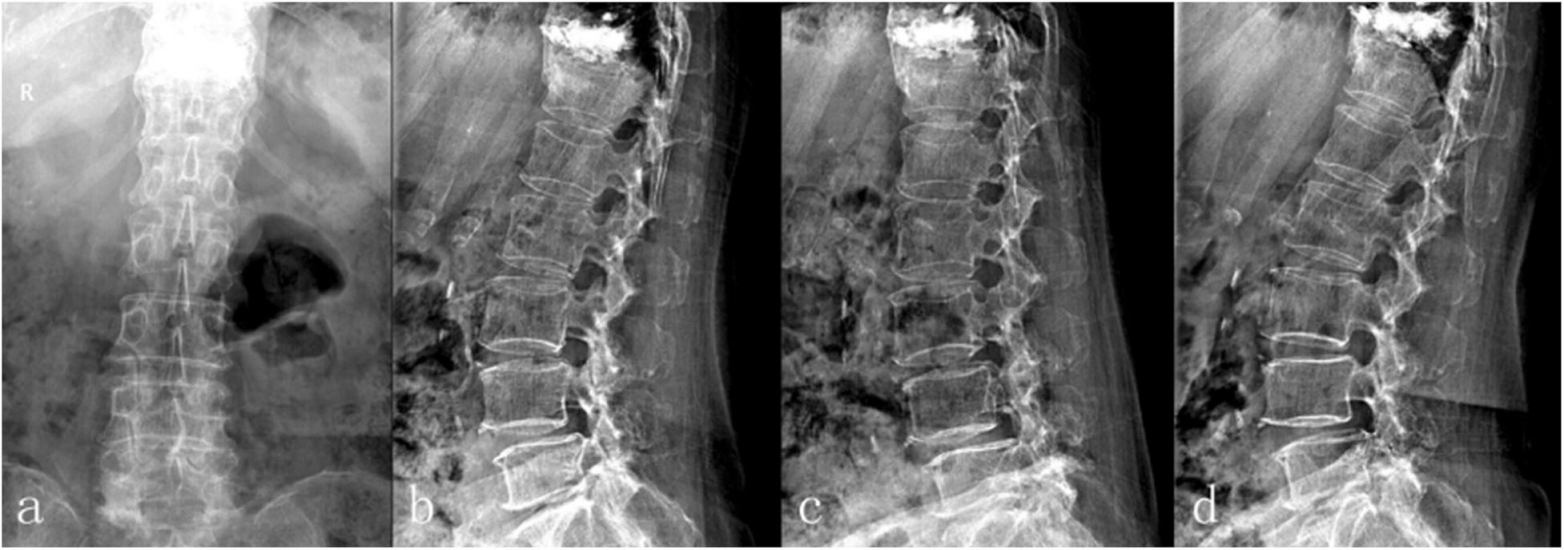
DR detected grade I degenerative spondylolisthesis with relative stability at L4 level with cement deposit at fractured T11 body. **a**: anteroposterior. **b**: neutral. **c**: flexion. d: extension

Digital radiographic (DR) images revealed grade I degenerative spondylolisthesis without any obvious instability at L4 level (Fig. 1).

**Fig. 2:**
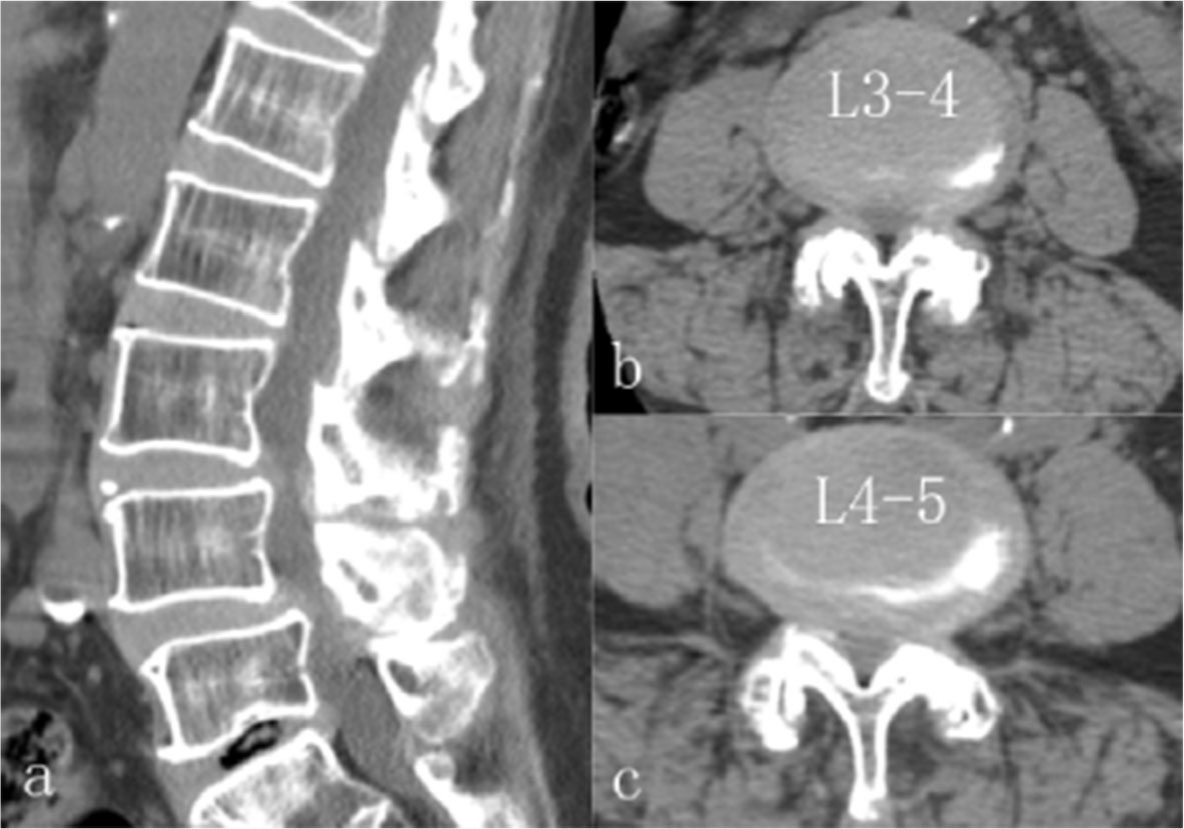
CT examination revealed degenerative spondylolisthesis at L4; moreover, LDH at L3–4 and L4–5, the latter was more severe, especially on left side

A CT scan indicated LDHs at L3–4 and L4–5, with the L4–5 herniation being much severer, particularly on the left side (Fig. 2). Her diagnosis was determined to result from LDH (L3–4, L4–5) and degenerative spondylolisthesis (L4–5, grade I).

### Operation

**Fig. 3:**
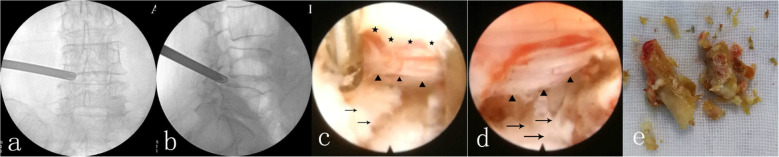
PTED at L4–5 level via left lateral approach. **a**: anteroposterior fluoroscopy. **b**: lateral fluoroscopy. **c**: the hypertrophic ligamentum flavum (stars) and herniated discs (arrow) were removed partially, transverse nerve (triangle) was carefully and well exposed. **d**: the dura sac was completely decompressed, and the traversing nerve root was floating freely. **e**: the removed fragments of herniated disc and ligamentum flavum

Stated thus, we concluded that the L4–5 lesion was the primary lesion causing her pain, while that in L3–4 was secondary. PTED surgery [6] was performed at the L4–5 level under local anesthesia (Fig. 3), while L3–4 remained untouched. In case this operation failed, the operation of L3–4 level would be performed. PTED procedures mainly consisted of an ectomy of the herniated disc and the removal of hypertrophic ligamentum flavum and facet joint osteophytes.

**Fig. 4:**
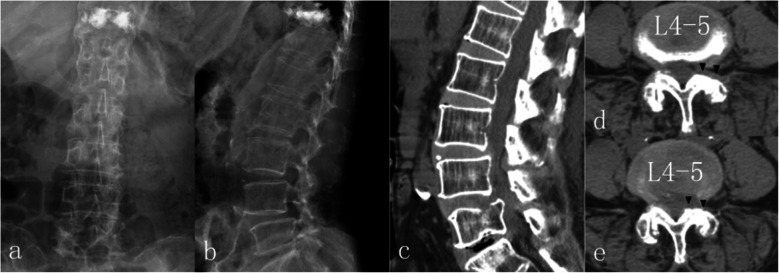
DR 3 days postoperatively demonstrated no slippage deterioration at L4–5 level (**a**-**b**). Postoperative CT re-examination detected anterior portion of superior articularis of L4 was removed, as well as the partial of L4–5 disc (**c**-**e**: triangle)

The day after PTED, the patient reported decreased pain in the lower back (VAS: 3) and limbs (VAS: 2) and improving strength. Three days later, her pain recurred (lower back pain VAS: 4; limb radiating pain VAS: 3), and a CT re-examination detected vestiges of the removed ventral bone of the responsible superior articularis and reduced disc, with a widened lateral recess and growing vertebral canal. The CT also showed an endplate injury of L5 upper end plate (Fig. 4). She was discharged 1 week later with improved symptoms.

### Postoperative course and managements

Two weeks postoperatively, during telephone follow-up, she complained of recurrent back pain (VAS: 4) without any worsening of the radiating pain (VAS: 3), though seen as NPF during early recovery stage. Consequently, NSAIDs and neurotrophic drugs were administered, and she was told to limit her activities for half a month; this intervention worked and her VAS decreased to 3. Subsequently, the dose of NSAIDs was reduced.

**Fig. 5:**
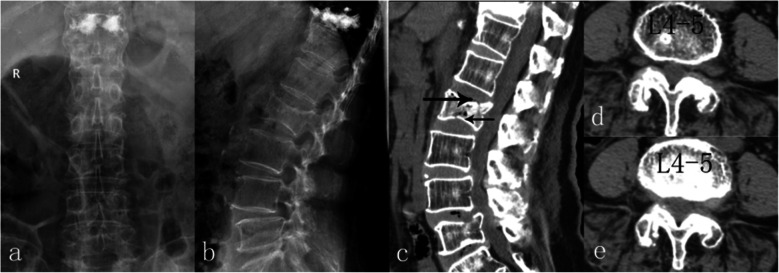
DR 8 weeks postoperatively revealed shortened height of L2 (**a**-**b**). CT examination detected a fresh comminuted fracture of L2 vertebral body; the irregular sharp fracture lines (arrow) indicated acute or subacute fracture (**c**). Transverse images at L4–5 level (**d**-**e**) revealed no reherniation of disc

However, 8 weeks postoperatively, the pain in her lower back recurred gradually and progressively, without relapse of her radiating leg pain, while neither accident nor obvious trauma could be recalled as a probable cause. Over the course of a week, her back-pain VAS reached 8 and NSAIDs and neurotrophic drugs no longer had any effect. She could hardly turn over in bed or stand or walk by herself, therefore prompting another hospital visit. This time, a physical examination revealed pain on percussion at L1–3 (++) and longitudinal percussion pain (++). DR images were obtained. To our surprise, the height of L2 vertebral body was decreased, apparently indicating OVF, was confirmed afterwards by CT (Fig. 5). Since she could not recall any injury history, it was deemed a latent osteoporotic fracture.

**Fig. 6:**
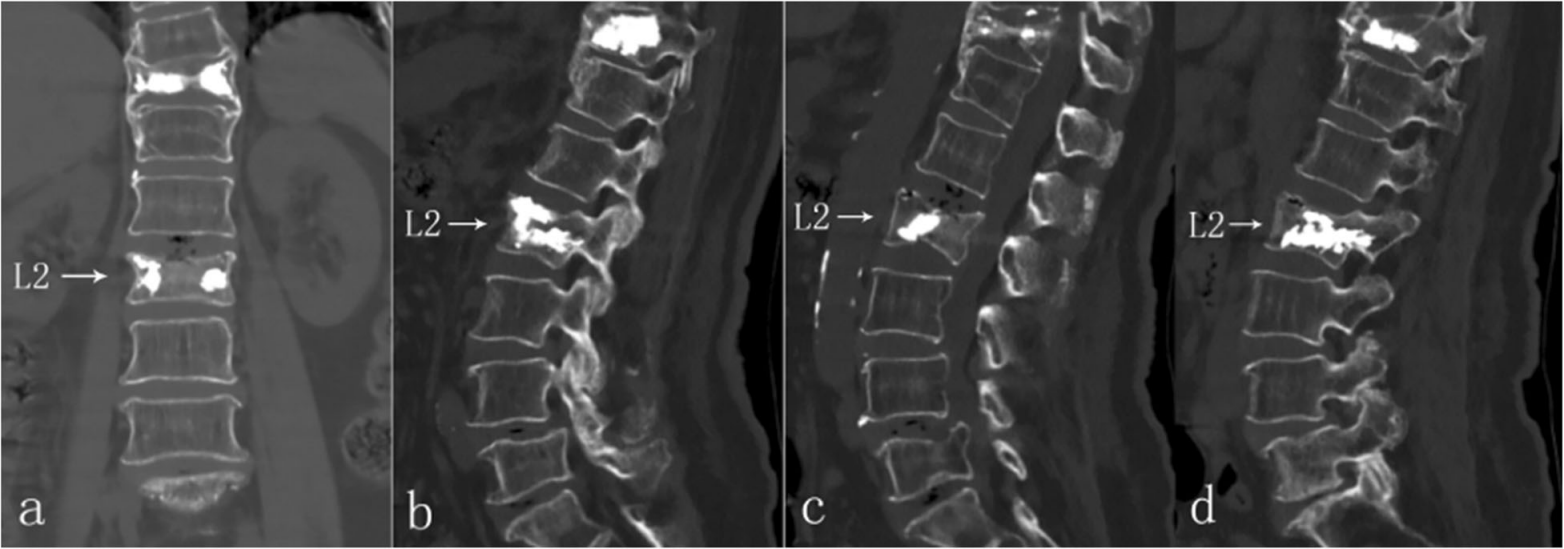
CT scan after L2-PVP illustrated no cement leakage with maintenance of vertebral height. (**a**-**d**. **b**: sagittal plane through left pedicle. d: sagittal plane through right pedicle)

PVP of L2 was performed under local anesthesia. The next day, she could walk in the ward with the help of a waist brace support. The VAS of her back pain decreased to 1. Repeat DR and CT examinations confirmed height restoration of L2 without cement leakage (Fig. 6). Bone mineral density (BMD) exam of her waist by dual energy X-ray absorptiometry was T < − 2.5 SD in the lumbar region; therefore, intensive anti-osteoporosis treatment, including drugs and rehabilitation, were advised.

**Fig. 7:**
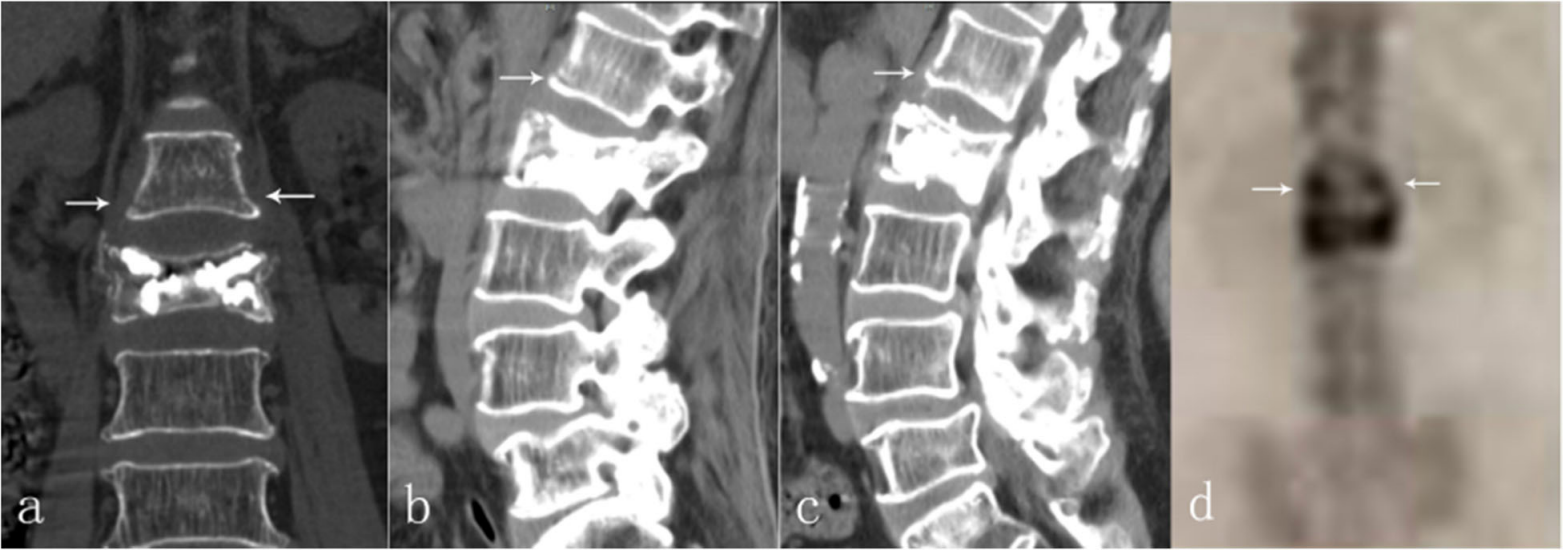
CT examination 2 weeks after L2-PVP detected an ambiguous fracture line at L1 (**a**-**c**, arrow), later reconfirmed by bone scintigraphy as fresh vertebral fracture (**d**, arrow)

Ten days following L2-PVP, the patient felt recurrent back pain after a severe cough, with a sudden increase in her VAS score to 7. Bed rest and pain-killers offered no relief. A CT re-scan revealed an ambiguous fracture line at the anteroinferior region of L1 which we had not noticed in the previous CT. Because of her history of electrode implantation, an MRI scan was deemed unsuitable for her. Bone scintigraphy confirmed a fresh fracture of L1 (Fig. 7).

**Fig. 8:**
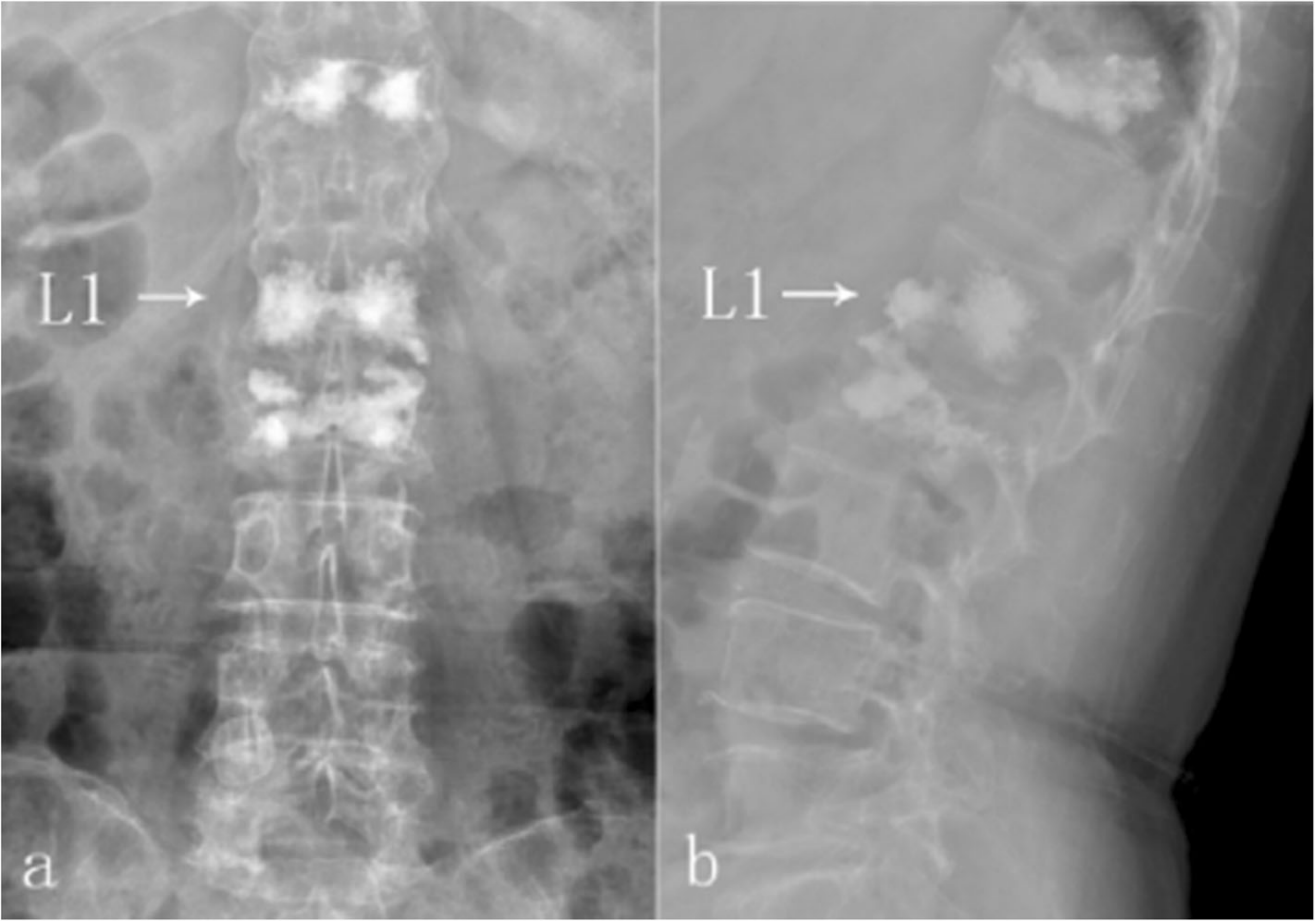
PVP of L1 (**a**-**b**, arrow)

PVP of L1 was performed and back pain disappeared immediately again, bringing down the VAS score to approximately 1 (Fig. 8). The back pain vanished without recurrence, with the SF-36 score adding up to 353 and JOA 25 points at 14 weeks postoperative following the PTED of L4–5. In addition, she scored 374 points on the SF-36 score and 26 points on the JOA at 36 weeks postoperatively. She could then walk freely and quickly.

## Discussion and conclusion

PTED for spinal disorders firstly was introduced in 1992 [7], the advantages of which include small incision, quick recovery, and equivalent or superior clinical outcomes compared with open surgery. With new emerging technologies, PTED variants have been widely developed [8], and are well accepted currently by surgeons and patients for their ease of use and simplicity [6 above]. Usually, a foramen approach is selected for L1-L5 LDH, and an interlaminar approach for L5/S1 LDH. However, fracture following PTED rarely happens and has been seldom been reported in the literature. The novelty and complexity of this rare case should attract attention.

Although PTED is advantageous in terms of its shorter recovery time and lower invasiveness in spinal surgery, it does not completely eliminate all symptoms once and for all. Some patients require a certain period of time for recovery. There are two hypotheses for the mechanism of recovery and its duration [4]: (1) pathoanatomy: for example, pain sensation (mainly conducted via C fibers lacking myelin sheaths) recovers sooner than numbness and paresthesia (both mainly from myelinated A-delta fibers), so a patient following PTED still feels pain in the recovery stage; (2) pathophysiology: due to mechanical compression or chemical irritation/inflammation, sensory discomfort might last long and be neither quick nor easy to eradicate. It takes time, on one hand, for demyelinated and/or dead nerve cells and fibers to regenerate and grow, and, on the other hand, for inflammatory irritation to cease. Therefore, this is a usual phenomenon, and a common consideration for the surgeons performing PTED is that the lower back pain and leg radiating pain sometimes would last or recur in this period of the recovery stage, for approximately 3 to 8 weeks. Symptoms such as pain, stiffness, swelling and other discomfort mostly would be NPF, and NSAIDs and physical factor therapy are of great help. However, 2 months later, the back pain persisted or even worsened, which may be persistent radicular pain due to incomplete decompression or OVF rather than NPF and post-operative dysesthesia. As a result, it has always confused us whether the pain reported by the patient in the recovery stage was NPF or a new extra injury. We consider the time period as the reason associated with this, for our experience more than trial evidence, within 2 months or so. In this case, an osteoporotic fracture was beyond our expectations, which explained the recurrent pain.

The disappearance of radiating pain to the leg [9] supported our preoperative assessment and PTED surgery plan in this case. However, we misunderstood the recurrence of her lower back pain as being caused by a lesion in the surgical site, or due to the regeneration of nerve fibers, neglecting it as NPF, finally delaying a correct diagnosis. If we carry on thinking of the recurring pain as NPF, the fracture would not heal and the patient would not be able to stand up or walk in the future. Moreover, conducting a telephone follow-up rather than performing a physical examination was another error on our part. This underscores the importance of conducting physical and imaging examinations in case traditional conservative treatment fails to improve symptoms following PTED.

The day after PTED, the patient reported decreased low back pain and improving strength, which probably encouraged the patient to do some daily activity, namely putting her at risk of idiopathic fracture. Cough or idiopathic osteoporosis may be another cause of fracture.

The main symptoms of PD are resting tremor, progressive rigidity, slow bradykinesia, and unstable posture. Unstable posture, clumsy movement, and poor coordination might predispose multiple falls. In our case, OVF at T11 after a fall accident 5 years ago might be an indication of the deterioration of PD. DBEI was another hint indicating PD severity. Patients in the progressive stage have reduced mobility, poor appetite, aging, which lead to bone mass reduction and osteoporosis.

Osteoporosis, along with the abovementioned factors, might increase risk of OVFs, compared to that in the general population [5]. Lee et al. [10] pointed out that age over 65, female gender and low income may be significant factors for PD, as well as Osteoporosis and OVF, which could have happened without any radicular symptoms or trauma history. Furthermore, PD patients are more likely to develop OVFs, especially at the advanced stage of the disease, without necessarily having a clear trauma history. Even a normal daily activity could be a cause of OVF.

Perhaps in the past, the patient had adapted to frequent multiple falls and various other kinds of small trauma happened constantly as normal daily incidents, such as cough or lifting a slight object. She could not recall what caused the L2-OVF, explaining the poor osteoporosis condition on BMD. Besides the multiple fractures and DXA results, L1-OVF after a severe cough indicated that the patient had developed severe osteoporosis. So these clues should have been given more attention. Aggressive medical treatment using teriparatide (Forteo), Calcitriol, or a combination with denosumab (Prolia), and Vitamine-D3-calcium supplements, trunk protection, and various types of anti-osteoporosis treatments was recommended when discharged. We also suggested the patient to not overdo activities after both PTED and PVP, and to protect herself with a brace to ensure soft tissue injury restoration, reduce inflammation and facilitate fracture union.

For complex cases with multiple OVFs, the computed tomography Hounsfield unit (CT HU) is of great value in evaluating the BMD [11]. MRI lipid suppressed and diffusion weighted imaging are gold standards in an OVF diagnosis; however, this patient underwent DBEI for PD. We consulted the engineer involved when the DBEI was first placed and were informed that the magnetic solenoid field would severely impair DBEI, potentially leading to a destructive thermal burn. Hence, MRI was not suitable for her. Therefore, bone scintigraphy and CT HU might be a supplementary plan of MRI in checking spinal fracture.

To conclude after all, we firstly report an unusual case of recurring pain following PTED misdiagnosed as NPF. In elderly patients with existing osteoporosis and especially PD, it would be best to first check for any fractures. Besides, a relapse of back pain of PD patients following PTED, with no improvement after conservative treatment, may indicate that OVF and should not been taken for granted as NPF. In such instances, a further physical examination and imaging check-up are warranted. Furthermore, bone scintigraphy is an alternative to examine fractures in these extremely complicated cases.

## Data Availability

All data and information on this report are available on request from the authors. All rights of the submitted article is to be transferred and assigned to BMC Musculoskeletal Disorders Company, for sole right to print, publish, distribute and sell in all languages and media internationally.

## Abbreviations

OVF: Osteoporotic vertebral fracture
NPF: Normal postoperative phenomenon
PTED: Percutaneous transforaminal endoscopic decompression
LDH: Lumbar disc herniation
NSAIDs: Non-steroidal anti-inflammatory drugs
PD: Parkinson’s disease
PVP: Percutaneous vertebroplasty
VAS: Visual analogue scale
DBEI: Deep brain electrode implantation
SF-36: Short-form-36-questionnaire score
JOA: Japanese orthopedic association score
DR: Digital radiographic
